# Retroperitoneal laparoscopic partial nephrectomy for treatment of metanephric adenoma (Report of 6 cases)

**DOI:** 10.1186/s40064-016-2662-y

**Published:** 2016-07-07

**Authors:** Ju Guo, Xiaochen Zhou, Bin Fu, Runfu Cao, Weipeng Liu, Gongxian Wang

**Affiliations:** Institute of Urology, First Affiliated Hospital of Nanchang University, Nanchang, China

**Keywords:** Metanephric adenoma, Retroperitoneal, Laparoscopic partial nephrectomy

## Abstract

**Objectives:**

To investigate the clinical and pathological features of metanephric adenoma (MA) and the clinical outcome after retroperitoneal laparoscopic nephron-sparing surgery.

**Methods:**

Six out of 183 partial nephrectomies performed during January 2009 to August 2014 were confirmed to be MA confirmed by postoperative pathological study. Perioperative parameters of the six patients were then retrospectively collected, analyzed and compared with current literature, including warm ischemia time (WIT), total operation time, estimated blood loss (EBL), positive surgical margin (PSM), and complications. Surgical and oncological outcome of all six patients were evaluated based on a mean follow up of 17 months (5–48 months).

**Results:**

Tumors in all six cases were all successfully removed by partial nephrectomy. Mean WIT was 24.7 min (19–35 min). Mean operation time was 103.6 min (82–147 min). Mean EBL was 53.5 ml (20–85 ml). No conversion, transfusion or other major complication were observed in all six cases. Postoperative pathology confirmed negative surgical margin in all six cases. During a mean of 17 month follow up (5–48 months), no local recurrence or metastasis were found in all six cases.

**Conclusion:**

MA is a rare benign primary kidney epithelial cancer, which could hardly be differentiated from renal malignancies based on preoperative imaging. Our data suggested that retroperitoneal laparoscopic partial nephrectomy can be used for surgical treatment of MA, in terms of tumor control and preservation of renal function.

## Background

Metanephric adenoma (MA) is a very rare benign tumor derived from kidney epithelial tissue, with less than 100 cases reported in current literature (Kuroda et al. 2003). The clinical symptoms and signs of MA are very similar to those of malignant renal tumors, which make the differential diagnosis and subsequent surgery planning a difficult task. Currently, surgery planning for MA is still identical to renal cell carcinoma, while the nature of the tumor can only be confirmed by postoperative pathological study (Kuroda et al. 2003). In this study, we reported 6 cases of MA confirm by postoperative pathological study. The purpose of this paper is to describe our preoperative imaging and postoperative histological diagnosis of MA in six patients, as well the surgical technique and oncological outcome based on a mean follow up of 17 months (5–48 months).

## Methods

### Patient selection

Six out of 183 retroperitoneal laparoscopic partial nephrectomy (RLPN) performed during January 2009 to August 2014 were confirmed to be MA confirmed by postoperative pathological study, including 4 females and 2 males (Table 1). Average age was 53.7 years (42–65 years). All 6 cases presented unilateral tumor, 3 on the right side and 3 on the left. Mean diameter of tumors was 3.7 cm (2.8–5.3 cm). 2 patients presented flank pain; 1 patient had concomitant bladder calculus and was administered due to urinary frequency and dysuria; the remaining 3 patients did not have any symptoms related to renal mass which was found during routine health examination. None of the patients presented hematuria.

### Imaging

**Fig. 1:**
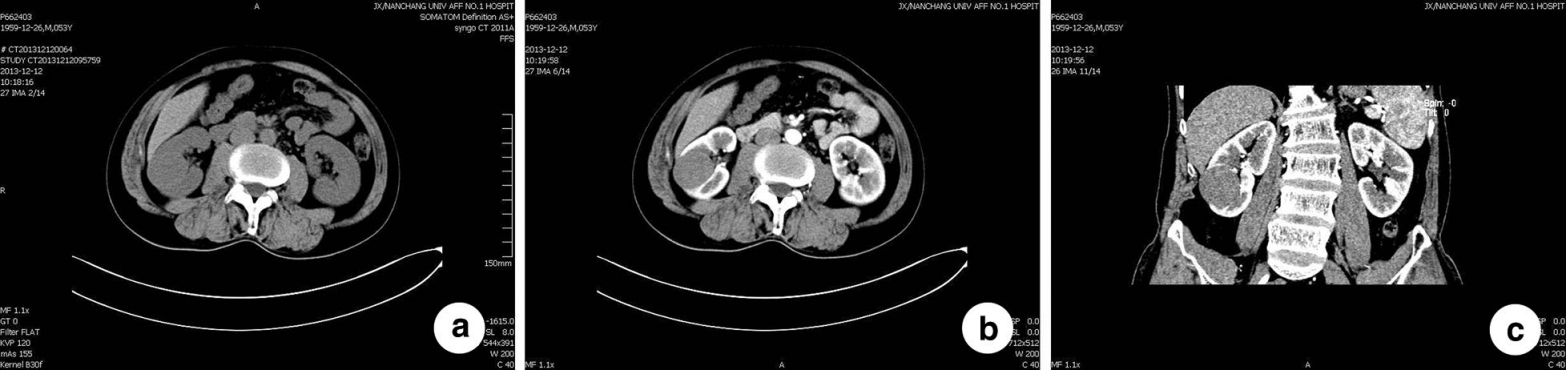
Preoperative CT scan. **a** Non-contrast CT scan results indicated that there was soft tissue density *shadows* in the lower-middle portion of the *right* kidney, with a clear boundary, a size of 4.2 × 3.8 cm and a CT value of 30–36 HU. **b**, **c** The density is uniform, and the enhancement degree of the tumor was significantly lower than that of the surrounding normal renal parenchyma

*CT* Non-contrast CT scan of 3 cases displayed soft tissue density shadow with capsule and blurred edge. The homogeneous density of the tumor was equivalent to, or less than the density of the renal parenchyma. A clear boundary could be observed to separate tumor from renal parenchyma. The other 3 cases showed a higher tumor density than renal parenchyma, with 1 case showing punctate calcification. Enhanced CT scan results indicated a heterogeneous and mildly progressive enhancement of the mass, but the degree of the enhancement of this mass was less than that of the adjacent normal kidney tissue. CT diagnosis indicated solid renal mass with high possibility of being malignant in 5 cases, while the remaining case was considered to be benign (Fig. 1).

### Treatment

**Fig. 2:**
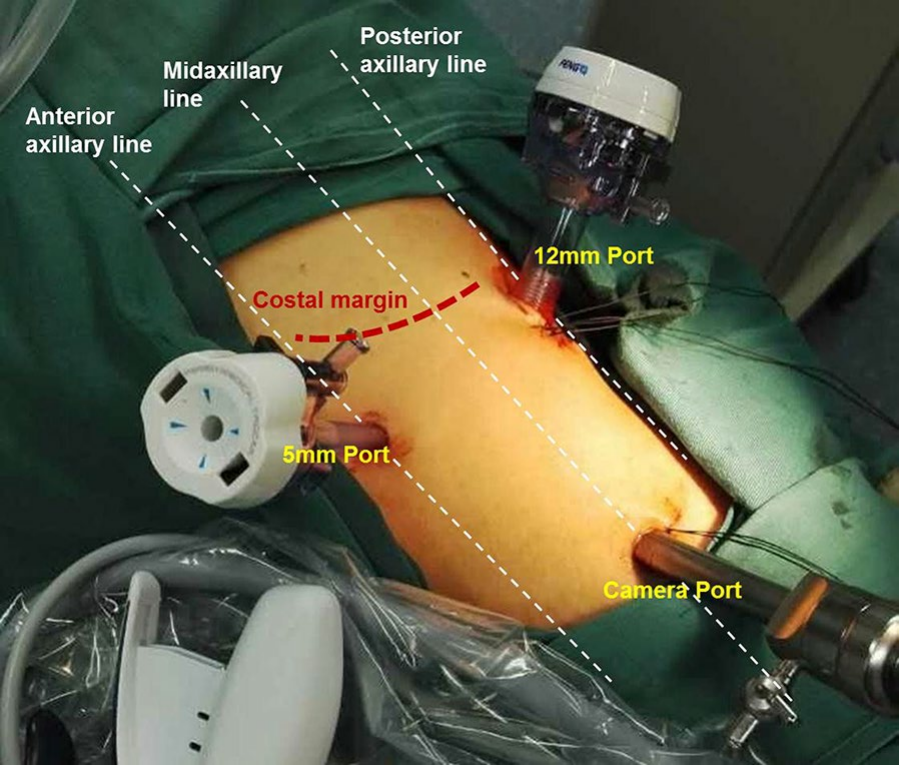
Trocar configuration for retroperitoneal laparoscopic partial nephrectomy

Tumors of all 6 cases were removed laparoscopically (RLPN) via an retroperitoneal approach. Surgical procedures:

After general anesthesia and routine intubation, patient was placed in 90 degree lateral position with the affected side facing up. Trocar configuration was shown as Fig. 2: with the camera port placed at two finger breath above the iliac crest. The retroperitoneal space was created by Hasson open technique with a balloon dilator. Pneumoperitoneum pressure was maintained at 12–15 mmHg (1 mmHg = 0.133 kPa) when all ports were placed and properly sealed. After removing the retroperitoneal fat, Gerota fascia was identified and opened longitudinally. Dissection of the kidney was carried on within Gerota fascia until the tumor was completely exposed. Renal artery was subsequently identified by arteriopalmus near the hilum, which was controlled by laparoscopic bulldog clamps. The tumor was then dissected from the parenchyma together with a 0.5 cm normal tissue. If the tumor had a clear capsule, the capsulated tumor was then removed while keeping the capsule intact. After the tumor was removed, renal parenchyma was closed in two layers with 5-0 V-Loc for the inner layer (collecting system) and 3-0 V-loc for the outer layer. Finally, blood flow was restored by removing bulldog clamp on the renal artery. Samples was removed after cautious inspection for any bleeders.

## Results

### Perioperative parameters

All tumors were successfully removed by RLPN without conversion or transfusion. Mean warm ischemia time (WIT) was 24.7 min (19–35 min). Mean operation time (OT) was 103.6 min (82–147 min). Mean estimated blood loss (EBL) was 53.5 ml (20–85 ml). Patients were discharged on day 7 (5–10 days) postoperatively.

**Fig. 3:**
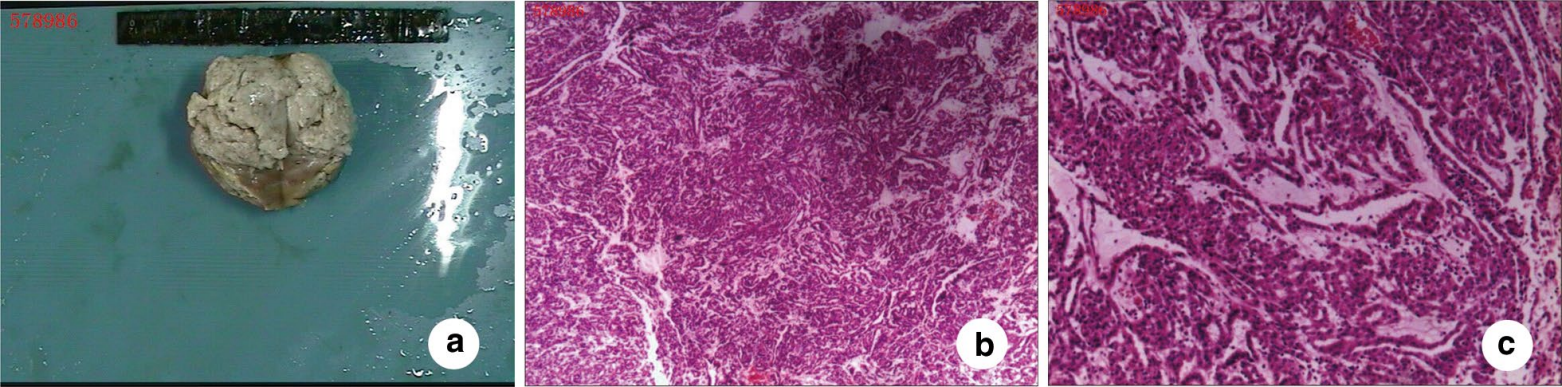
Pathological study. **a** tumor cross-section. **b**, **c** cells analyzed under microscopy showed the same size, with a relatively small volume, adenoid gland shape, small tubular arrangement, unclear nucleoli, no nucleus fission, and a loose and bright mesenchyme. (magnification: **b** × 4 objective; **c** × 10 objective)

Mean diameter of tumors matched with preoperative CT scan, with or without capsule. The cross-section of the tumor showed a light yellow or gray soft and homogeneous characteristic (Fig. 3a). Only 1 case displayed intra-tumoral bleeding and a limited necrosis, while another case showed various extent of calcification. Pathology confirmed all 6 tumors were metanephric adenoma (Fig. 3b, c). Positive surgical margin was found in none of the 6 resected tumors (Table 1).

**Table 1:** Patient data and perioperative assessment

Case number	Age (year)	Gender	Initial presentation	Laterality	Tumor size (cm)	WIT (min)	Operative time (min)	EBL (ml)	Postoperative hospital stay (day)
1	42	Female	Flank pain	Left	3.3	25	120	50	6
2	52	Female	None	Left	2.8	30	82	20	5
3	58	Male	None	Right	4.3	19	90	56	6
4	65	Female	Urinary frequency and dysuria due to concomitant bladder calculus	Right	3	24	108	55	8
5	47	Female	Flank pain	Right	5.3	25	147	85	10
6	58	Male	None	Left	3.6	25	75	55	

*WIT* warm ischemia time; *EBL* estimated blood loss

### Follow-up and prognosis

**Fig. 4:**
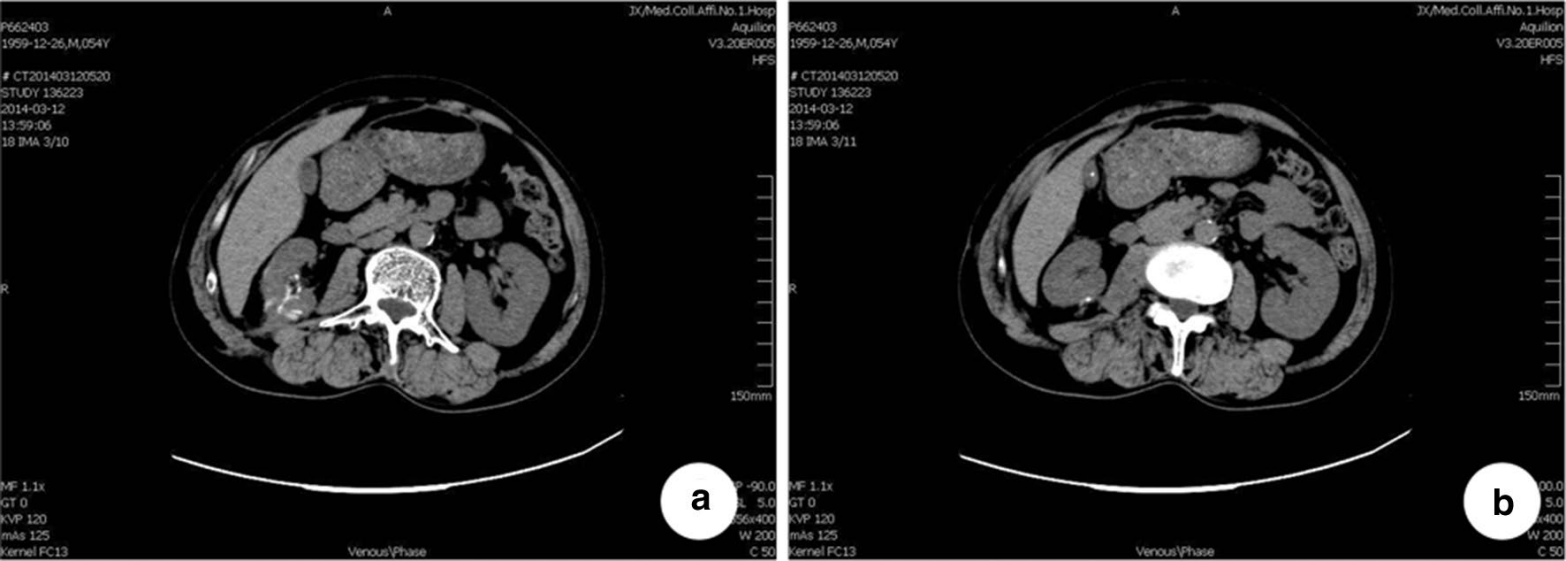
CT scan results of 4 months follow-up after surgery, **a** preoperative CT scan; **b** 4 month postoperative scan

During a mean of 17 month follow up (5–48 months), no local recurrence or metastasis were found in all six cases confirmed by CT (Fig. 4).

## Discussion

MA is a rare tumor, accounting for the 0.2 % of adult renal epithelial tumors (Kuroda et al. 2003), with only few reports in the literature. MA is usually considered as benign tumor because of its biological properties. In 1998, WHO classified it as one of the benign renal epithelial tumors, including renal oncocytoma and renal papillary adenoma (Torricelli et al. 2011). The disease can occur at any age, although appear to be more common in middle-aged women. The male to female ratio is about 1:2 to 1:3. MA does not show much difference regarding the common clinical signs and symptoms that are typical of renal malignancies, including lower back pain, hematuria, and the presence of a tumor mass. The majority of the patients do not show any symptoms or characteristic signs. Thus, MA can be found during a routinely examination or during the treatment for another comorbidity. Most of the patients presented lower back pain, and 2 patients showed hematuria symptoms, although not pronounced. It was reported that 12 % of the patients might show polycythemia, which was the most common presentation among all renal tumors (Davis et al. 1995). In our group of patients, only 1 displayed polycythemia. This may be associated with the renal adenoma cells that produce and secret erythropoietin and a variety of other cytokines (Yoshioka et al. 2007).

The diagnosis of MA based on history, physical examination and radiological studies remains a difficult task because of the similarity in symptoms and radiological appearance between MA and renal malignancies. Ultrasound is usually able to detect a single and remarkable mass emerging from the kidney surface or inside the renal parenchyma, displaying a hypoechoic, hyperechoic, and uniform echo (Zambrano et al. 2012). Color Doppler shows lack of vascular tumors (Patankar et al. 1999), with the mass around the bar to see color flow signals. CT scan allowed us to detect an equal density or a high density solid mass (CT value of 20 ~ 40 HU), clear boundaries, capsules, lack of uniformity of the internal mass, presence of hemorrhage in the internal mass in a small number of patients, as well as necrosis and calcification. CT scan showed that the tumor was slight and gradually enhanced in each period, with a lower enhancement degree than that of the adjacent normal kidney tissue. It is debatable whether to use needle biopsy for preoperative diagnosis (Ebine et al. 2004). We did not perform preoperative biopsy because of the following two reasons: 1) additional risk of tumor spread and bleeding; and 2) the surgical approach or planning was the same for either renal cell carcinoma or MA. We did not perform intraoperative frozen sectioning for the same reason.

MA usually appears as a unilateral single mass, and may resides in any part of the kidney, although more prominent in the renal cortex of the kidney surface, with no clear invasion, with or without a capsule. Some studies reported tumor diameter could range from 0.3 to 15.0 cm (Davis et al. 1995). Tumor section is of a yellowish or gray color, and homogeneous, which may have cystic degeneration, hemorrhage and necrosis, even calcification. Round and small tumor cells are visible under the light microscope, tightly configured as small tubular, or acinar, or solid shape, sometimes piled into spherical clumps like bud-like structures or neonatal glomerular cells, which represent a unique structure of MA useful for diagnosis and differentiation. They are mature, with atypical and rare mitosis.

Since MA is not characterized by specific and typical clinical symptoms and signs, imaging studies are facing a difficult interpretation. In addition, since MA is a rare tumor, it is easily misdiagnosed as kidney cancer. In the absence of clear pathological findings, clinical treatment and surgical methods are basically equivalent to the treatments for kidney malignancies, such as nephron-sparing surgery or radical nephrectomy (Ebine et al. 2004; Xu et al. 2012; Kumar et al. 2007).

The lack of sufficient MA clinical data prevents the improvement of its treatment with little literature support. Several studies reported the use of laparoscopic nephrectomy (Ebine et al. 2004), and only few cases of laparoscopic partial nephrectomy (LPN) for renal adenoma were reported (Xu et al. 2012; Kumar et al. 2007). Raunak et al. (Patel et al. 2013) reported one case of renal adenomas successfully removed using the robot-assisted LPN. The RLPN for renal adenoma that we described in this study has not yet been reported. The pre-operative diagnosis of the 6 patients enrolled for this study was of kidney cancer, according to the size of the tumor and other characteristics. However, the use of RLPN revealed a successful result of a complete removal of the tumor. Retroperitoneal operation approach is a feasible approach for smaller tumors in obsesses patients in Asian population (Wang et al. 2011). Ouzaid and others reported that (Ouzaid et al. 2012) abdominal surgery approach prolonged the intra-peritoneal route time and hospitalization. The transperitoneal approach is generally considered as an advantageous surgical method because of the “easy to find” renal artery to block it (artery in front, vein in the back). When the renal adenoma biological characteristics are typical of a benign tumor, RPLN is used for therapeutic purposes, avoiding the misdiagnosis leading to an unnecessary nephrectomy, especially for T1b pre-operative diagnosis of possible malignant renal mass. The 6 patients underwent RLPN successfully, with the preservation of the renal function, and no sign of tumor recurrence and distant metastasis during the follow-up.

In conclusion, MA is a rare primary renal tumor, with no distinct clinical symptoms and radiological characteristics. It can be easily confused with malignancies of the kidney. RLPN is shown to be a safe and feasible approach for treatment of MA both oncologically and functionally.
